# The Evolving Global Epidemiology of Human Melioidosis: A Narrative Review

**DOI:** 10.3390/pathogens13110926

**Published:** 2024-10-24

**Authors:** Francesca F. Norman, Barbra M. Blair, Sandra Chamorro-Tojeiro, Marta González-Sanz, Lin H. Chen

**Affiliations:** 1 National Referral Unit for Tropical Diseases, Infectious Diseases Department, Ramón y Cajal University Hospital, IRYCIS, 28034 Madrid, Spain; 2 CIBER de Enfermedades Infecciosas, Instituto de Salud Carlos III, 28029 Madrid, Spain; 3 Health Sciences, Universidad de Alcalá, 28801 Madrid, Spain; 4 Division of Infectious Diseases, Massachusetts General Hospital, Boston, MA 02114, USA; 5 Harvard Medical School, Boston, MA 02115, USA; 6 Department of Medicine, Mount Auburn Hospital, Cambridge, MA 02138, USA

**Keywords:** *Burkholderia pseudomallei*, travel, imported, climate change, hemophagocytic lymphohistiocytosis, nosocomial transmission

## Abstract

Endemic in over 45 countries globally, recent reports of locally acquired melioidosis in novel geographical areas, such as the Southern US, have highlighted the expanding geographical range of *Burkholderia pseudomallei*. Climate change and severe weather events have been linked to an increase in cases of melioidosis, which follows environmental exposure to the bacterium. Healthcare professionals should be aware of the possibility of the disease, with its diverse and often delayed presentations, even in areas not previously known to have risk. Over 200 cases of travel-associated melioidosis have been reported in the literature, highlighting the need to consider this disease in non-endemic areas, as diagnostic delays of up to 18 months have been identified. The review updates the global epidemiology of melioidosis, focusing on new geographical areas where cases have been diagnosed and imported cases, unusual clinical presentations and co-infections, and less frequent modes of transmission (laboratory exposures and the risk of acquisition due to imported infected animals and contaminated products).

## 1. Introduction

The recent report of locally acquired melioidosis in the Southern USA highlighted the expanding geographical range of *Burkholderia pseudomallei* [1]. Officially present in around 45 countries worldwide, mainly in Asia, modeling studies suggest underreporting and much wider geographical distribution, with autochthonous cases described in Africa, America and the Caribbean [2,3].

Climate change and severe weather events have been linked to an increase in cases of melioidosis, which follows environmental exposure to the bacterium [4,5]. An outbreak of melioidosis occurring during two La Niña events in an Australian region previously considered non-endemic for *B. pseudomallei* was notified recently [5]. However, traditional risk factors may not always be present, as documented by another outbreak of localized cutaneous melioidosis in immunocompetent children in Australia following a sporting event during the dry season [6]. In the context of the recent COVID-19 pandemic, new scenarios have also arisen, as illustrated by an outbreak of acute melioidosis in patients with SARS-CoV-2 infection linked to contact with contaminated tap water in a field hospital in Thailand [7].

The role of population movements in the epidemiology of infectious diseases must also be considered. Two reviews on travel-related melioidosis spanning 1982–2022 analyzed over 200 cases and presented a picture of the main characteristics of imported infections, mainly acquired by males (>70%), in Asia (>70%), with pneumonia and sepsis as the most frequent presentations and diabetes mellitus and chronic lung disease as frequent co-morbidities [8,9]. These reviews noted both acute and latent presentations and emphasized the increasing identification of melioidosis in areas not previously associated with high risk; around 15% of travel-related infections were acquired in the American continent/Caribbean area and in Africa. Additional infections in travelers have been reported in 2023 and 2024 [10,11]. Risk groups such as immunocompromised patients merit special consideration, and expert recommendations relating to regionally limited bacterial infections like melioidosis in hematopoietic stem-cell transplantation donors and recipients have recently been updated [12].

Healthcare professionals should be aware of the possibility of the disease, with its diverse and often delayed presentations, even in areas not previously known to have risk. Lack of clinical suspicion may have further consequences with the inadvertent exposure of individuals such as laboratory personnel, leading to evaluation for post-exposure prophylaxis and extensive contact tracing and monitoring [13].

The review updates the changing global epidemiology of melioidosis to enhance awareness among healthcare professionals, focusing on new geographical areas where cases have been diagnosed and imported cases, unusual clinical presentations, data on special populations such as immunosuppressed patients, co-infections and less frequent modes of transmission (laboratory exposures and risk of acquisition due to imported infected animals and contaminated products).

## 2. Updates in Global Epidemiology

Melioidosis has classically been described in Southeast Asia and Northern Australia [14]. The disease has been spreading to new geographical areas in Asia, and it has also affected other continents, such as Africa and America [15,16,17,18]. *B. pseudomallei* originated in Australia, with subsequent introduction into Asia, and anthropogenic factors may have assisted in *B. pseudomallei* dissemination to Africa. Phylogenomic analysis of isolates has shown that African strains probably originated from Asia and were related to South American strains, possibly reflecting a recent shared evolutionary history [19].

Recently, cases of melioidosis have been reported in the US Gulf Coast of Mississippi in patients with no travel history, where the acquisition probably followed environmental exposure as the bacterium was found in soil samples [1]. This has triggered the Centers for Disease Control to consider melioidosis an endemic infection in the US [20,21]. Other Public Health interventions highlighted the need to strengthen disease awareness and diagnostics, emphasizing the risk of misdiagnosis with some microbiological platforms [21,22]. Public Health preparedness also includes surveillance, building laboratory capacity (biosafety level 3 is required) and developing training and protocols; adequate financial support must be allocated to perform these activities [22].

Melioidosis should also be considered in patients with suggestive clinical features who have traveled to any of the novel geographical areas [22]. Regarding imported melioidosis, previous reviews up to 2015 [9] and from 2016 to 2022 [8] showed that the most frequent regions of acquisition and diagnosis were Asia (mainly Thailand) and Western Europe, respectively. Table 1 summarizes the previous literature reviews on travel-associated melioidosis and outlines the main characteristics for ten additional cases reported from 2023 to mid-2024 [8,9,10,11,23,24,25,26,27,28,29,30]. These cases were identified following a search in PubMed (https://pubmed.ncbi.nlm.nih.gov, accessed on 30 June 2024) and Google Scholar (http://scholar.google.com, accessed on 30 June 2024) for articles in English using the terms melioidosis and *Burkholderia pseudomallei* (January 2023–June 2024). These ten most recent cases were also mainly acquired in Asia (8/10, 80%), with one case linked to exposure in Africa (Mauritania) and another in Central America (Honduras). Diabetes was the main identified risk factor (7/10, 70% and one other patient with pre-diabetes), although one case had no known risk factors. Due to the geographical expansion of the distribution of the disease and the increasing trend in international travel, imported melioidosis is being reported worldwide with novel geographical areas featuring as countries of acquisition [31].

## 3. Clinical Manifestations Including Less Usual Presentations

Melioidosis can manifest with wide-ranging clinical presentations in patients living in endemic areas as well as travelers, most frequently with pneumonia [8,32,33,34]. Over half the cases have bacteremia, and 20% develop septic shock. Infection can involve any body site and organ, including abscesses on the skin and soft tissues, in the liver or spleen, genitourinary tract infections, bone and joint infections, encephalomyelitis, meningitis or brain abscesses, gland abscesses such as parotid, and bacteremia without focal site. Skin infections were commonly reported, especially among children in Australia, usually occurring as a single abscess without sepsis [32,33]. In some Southeast Asian countries, parotid abscesses are the most common presentation in children [32]. Genitourinary sepsis with prostatic abscesses is especially common in males in Australia, and female genitourinary tract infections also occur [32,33,35].

A 30-year analysis of 1148 patients diagnosed with melioidosis in an endemic area at the Royal Darwin Hospital identified the majority of cases to be acute (88%), while 9% were chronic and 3% were reactivation; among deaths, 12% were attributable to melioidosis. Pneumonia was the main diagnosis in 52% of cases, skin abscesses in 13%, and genitourinary infection in 12% of cases. The latter involved mainly males (74%) who had prostatic abscesses. Eleven percent of cases presented with bacteremia without a focal site of infection, and these tended to be immunocompromised patients. Children were far more likely to present with skin abscesses compared to adults (58% vs. 11%) and less likely to have bacteremia compared to adults (15% vs. 58%) [33].

Separately, a prospective multi-center cohort study of 1352 patients from northeastern Thailand, a highly endemic region, also found lung infection to be the most common presentation (42%), which is associated with increased risk for death. Sixty percent of all deaths in one month can be attributed to lung infection. Skin and soft tissue infections were less common (23%) and had lower mortality at one month (14%). In this cohort, *B. pseudomallei* bacteremia was present in 77% [34].

Among travel-related cases, the frequently reported presentations included pneumonia (35%), sepsis (29%), skin/soft tissue infections (14%), genitourinary involvement (including prostatic abscess) (11%), CNS involvement (9%) and endovascular infections (mycotic aneurysms, prosthetic valve infections, pericardial effusion) (6%) [8].

### 3.1. Pediatric Melioidosis

Most studies and reviews that described pediatric melioidosis cases generally found only a small proportion to occur in children and with a higher proportion of skin and soft tissue infections in children compared to adults [6,32,33]. Although invasive disease can also occur in children, *Burkholderia pseudomallei* infection in children can be subclinical; some evidence from a seroprevalence study in Thai children aged <15 years estimated symptomatic disease to occur only in 1 out of 4600 antibody-producing exposures [36]. The most frequent cutaneous manifestations are solitary abscesses [32,33], but multiple pustules and cellulitis also occur, and erythema nodosum has been reported in children with melioidosis, all involving lower limbs [37,38,39].

Head and neck involvement of melioidosis has been reported frequently in children [40,41,42]. Among 34 children with culture-confirmed melioidosis in Sarawak, Malaysia, 59% had an infection located on the head or neck [42]. Infected sites include cervical lymph nodes (95%), salivary gland (25%), and, less commonly, lacrimal gland (dacryocystitis) and scalp abscesses. An analysis describing a 10-year experience (2009–2018) with 355 children with culture-confirmed melioidosis in northern Cambodia reported that parotitis was the most frequent presentation, found in 27% of the cohort. In this pediatric population, the in-hospital case fatality rate was 11.5%; risk factors for death included bacteremia, pneumonia, female sex, and age <5 years [40]. Notably, this study reported that some children (n = 39) recovered after treatment solely with oral antimicrobial drugs, which suggests a milder course in some pediatric melioidosis patients [40].

Another recent retrospective study from Vietnam analyzed 35 culture-confirmed melioidosis in children <16 years of age between July 2015 and August 2019. This series also found suppurative parotitis to be the most common presentation (43%), followed by lung infection (29%) with disseminated disease, septicemia (20%), and less commonly central nervous system symptoms (11%); case fatality was still high at 11% [41].

In a systematic review that identified 22 cases of neonatal melioidosis (acquired through mother-to-child transmission, healthcare-associated or community-acquired infections), *B. pseudomallei* sepsis and/or pneumonia and/or meningitis were the most frequent diagnoses, with clinical presentations including fever and respiratory distress but also nonspecific symptoms such as poor feeding, lethargy, and grunting [43].

### 3.2. Genitourinary Manifestations

In adults, pneumonia is the most frequent presentation of melioidosis, but genitourinary infections are also commonly reported in patients living in endemic areas and also in international travelers, including prostatitis, prostate abscess, urinary tract infection [9,29,35,44,45]. Among 514 patients with 561 episodes of melioidosis from the Northern Territory, Australia, 70% of the patients were male [46]. Prostatic abscesses were identified in 21% of these episodes, usually by computerized tomography. Another series of 144 culture-confirmed melioidosis cases in men in Far North Queensland, Australia, from January 1998 through June 2017, found that all patients with prostatic abscess (n = 22, 15%) were adults aged> 18 years. The majority of patients with prostatic abscesses had symptoms such as dysuria, urgency, frequency, retention or incontinence, or positive urine culture for *B. pseudomallei*, but prostate tenderness was present in less than half [44,46]. Relapse of prostatic abscess is common; hence, surgical drainage is important for many of these patients [44].

Genitourinary infections in women have also been reported, though less frequently than in men [35]. Among 453 cases of melioidosis in Far North Queensland, Australia, evaluated between January 1998 and April 2023, 9% of women (13/140) had genitourinary involvement compared to 24% in men. The majority of these women (85%) also had other organ involvement, and two with only genitourinary tract disease had pre-existing anatomical defects. Genitourinary involvement was identified only with computed tomography in 23% of the affected women, underscoring the need for early imaging to identify unexpected sites of disease [35].

Complications such as chorioamnionitis and premature delivery due to *B. pseudomallei* infection during pregnancy have been reported. In one instance, placental infection occurred despite prolonged ceftazidime therapy [47].

### 3.3. Cutaneous Presentations

Skin and soft tissue involvement in melioidosis is estimated to occur in about 13–14% of melioidosis cases, with some series reporting higher proportions (e.g., the Australian pediatric patients and one report on European travelers) [33,37,48]. The clinical presentation of cutaneous melioidosis ranges from localized dermatologic findings such as a single skin lesion without systemic symptoms (primary cutaneous melioidosis) to multiple pustules as a result of the bacteremic spread of *B. pseudomallei* to the skin (secondary melioidosis) [49]. The manifestations are widely varied, including ulcers, abscesses, cellulitis, pustules, boils, and carbuncles, and less frequently, erythema nodosum, polyarteritis nodosa, Sweet syndrome, ecthyma-like lesions, necrotizing fasciitis, and extensive skin pustular eruption with sepsis [37].

A summary of 43 cases of cutaneous melioidosis (CM) from the literature published before January 2018 found that the majority (67.4%) were travelers, with nearly half returning from Thailand. Most of the resident cases occurred in China. The majority of this cohort (88%) had primary CM, including 30% with chronic infection. Half of the primary CM patients had only skin lesions, and the other half also had additional sites of involvement. The median incubation time was 3 weeks.

The skin manifestations were similar between travelers and residents, most commonly with skin abscesses (58%), followed by cellulitis (26%) and skin ulceration (21%). Infection most frequently involved legs and feet, and less commonly, head and neck (22%), arms and hands (19%), trunk (11%), and disseminated infection (5%). Among the secondary CM patients, abscesses were common (60%), one patient had inflammatory leg swelling (20%), and one had disseminated pustules (20%). All secondary CMs presented with an acute form. About a third (37%) underwent surgery. Death was reported in less than 5% [49].

### 3.4. Hemophagocytic Lymphohistiocytosis (HLH)

At least five cases of HLH, a severe inflammatory syndrome due to the excess activation of macrophages and T cells, complicating melioidosis, have been reported in the literature [50,51,52,53,54]. These cases were reported mainly from endemic areas (two cases from India, one from China and one from Australia), with one case diagnosed in New Zealand but probably acquired in Brunei. Two of these cases were fatal, and the other three patients survived (although one of the latter, occurring in a primigravida, medical termination of pregnancy was performed due to severe intrauterine growth restriction of the fetus).

Recognizing this potentially life-threatening condition, which may be triggered by various viral and bacterial infections, hematological malignancies and connective tissue diseases, among others, is essential, as the early initiation of specific therapies such as high-dose corticosteroids may be necessary in addition to the required antibiotics.

## 4. Melioidosis in Special Populations

The main predisposing risk factors for melioidosis are diabetes mellitus, hazardous alcohol intake, chronic renal and pulmonary disease, and immunosuppression, although a direct association between HIV infection and the risk of melioidosis has not clearly been established [55]. In the future, the occurrence of melioidosis in special risk groups may increase; the 30-year investigation of over 1100 patients with culture-confirmed melioidosis in northern tropical Australia found that over the three decades, there was a significant increase in the proportion of patients over 50 years of age, females, with diabetes, malignancy, and immunosuppression, whereas there was a significant decrease in patients with no risk factors [33]. This study found that 10% of patients had underlying malignancies and 9% received immunosuppressive therapy/had other forms of immunosuppression, and associated mortality in these groups was 18% and 17%, respectively, similar to that reported for some of the other risk groups, such as patients with rheumatic heart disease/congestive cardiac failure or chronic renal disease [33]. Another study from an endemic region in Thailand, analyzing the characteristics of 1352 patients with culture-confirmed melioidosis, found that although most patients had risk factors for the infection, patients without identified risk factors did not have a decreased risk of death, highlighting possible gaps in the knowledge of the pathophysiology of the disease [34].

### 4.1. Solid Organ Transplant Patients

There are several cases of melioidosis in solid organ transplant recipients (SOTR) reported in the literature. Most of these reports are from Australia and Southeast Asia, especially India [56,57,58,59,60]. In addition, of the cases reported, many are in renal transplant recipients who also had concomitant diabetes mellitus, a known risk factor for melioidosis [61]. The most common clinical presentation is an acute febrile illness with pneumonia, yet as the nickname given to melioidosis as “the great mimicker” suggests, there are many other clinical syndromes attributable to it, including visceral abscesses involving the liver, spleen, kidney and prostate; septic arthritis; and osteomyelitis [62]. Further, the immunosuppression associated with organ transplantation can reactivate latent infection with *B. pseudomallei*, posing a significant problem [62]. Thus, it is recommended that patients living in or from melioidosis-endemic regions or those with evidence of past exposure to *B. pseudomallei* should be screened for melioidosis prior to solid organ transplantation [62,63]. Additionally, recommendations for preventing exposure to *B. pseudomallei* are increasingly important, especially for solid organ transplant recipients residing in or visiting endemic areas by limiting exposure to soil and surface water by avoiding gardening and other risk activities during the wet season and wearing protective foot-wear and protective gear during such activities [62,63]. Trimethoprim–sulfamethoxazole (TMP-SMX), which may protect against melioidosis and prophylaxis, especially in the early post-transplant period or times of increased immunosuppression, could be considered in high-risk situations [62]. More importantly, melioidosis needs to be included in the differential diagnosis of fever of unknown origin in transplant recipients who have traveled to endemic areas, especially since diagnosis can be difficult if not considered [62]. Once diagnosed, treatment of melioidosis in the SOTR is accomplished in two phases. First, an intensive phase with a beta-lactam, usually ceftazidime or a carbapenem such as meropenem, is given for at least 14 days but up to 8 weeks if severely ill, followed by an eradication phase with TMP-SMX or doxycycline for at least 3 months [57,58,62,63]. *B. pseudomallei* is an important and likely underreported emerging pathogen among organ transplant recipients. In order to prevent mortality, all clinicians, but especially those from non-endemic regions, need to be aware of its existence and have a high index of suspicion for patients originally from or with recent travel to endemic areas.

### 4.2. HIV

Although immunosuppression has been recognized as a risk factor for human melioidosis, data on HIV and *B. pseudomallei* co-infection are limited [55].

In a retrospective study in northeast Thailand, co-infection was detected in a minority (8/524, 1.5%) of adults with melioidosis, and both clinical presentation and acute outcomes were found to be similar in HIV-positive and HIV-negative patients [64].

In the recent series reviewing travel-associated melioidosis, only 2% of patients (3/137) also had an HIV infection [8]. Of these, two had favorable outcomes, and the patient who died was a diabetic migrant presumed to have a latent melioidosis infection that was reactivated by COVID-19 [65,66,67].

### 4.3. Malignancy

Co-existence of melioidosis and malignant neoplasms appears to be infrequent; rather, there have been reports of atypical presentations of *B. pseudomallei* infections in which malignancy was initially suspected or that were initially misdiagnosed as tumors, mainly with pulmonary involvement [68,69,70].

Several reports have notified cases of melioidosis in patients with cancer. Four cases of melioidosis, with an associated 50% mortality rate, in the context of febrile neutropenia in patients receiving chemotherapy have been published [71,72,73]. A non-diabetic renal transplant recipient from Thailand with a rare simultaneous presentation of localized pulmonary *Burkholderia pseudomallei* infection and primary pulmonary adenocarcinoma has also been reported [74]. Reported associations of melioidosis with hematological malignancies are rare and include an unusual description of the infection and hairy cell leukemia in two travelers returning from Thailand [75]. The review of imported melioidosis identified three patients with underlying malignancies; two had hematologic malignancies (Non-Hodgkin’s lymphoma and multiple myeloma) and survived, and one fatal case was also diagnosed with malignant undifferentiated carcinoma [8,76,77,78,79].

### 4.4. Other Causes of Immunosuppression

Data on *B. pseudomallei* infection in patients with other causes of immune suppression are scarce. A patient with psoriatic arthritis, living in an endemic area, who had received therapy with etanercept, ustekinumab and adalimumab was diagnosed with *B. pseudomallei* bacteremia following mild skin trauma while gardening after a period of heavy rainfall [80]. An uncommon presentation of severe melioidosis in a female with newly diagnosed systemic lupus erythematosus has also been reported [81].

*B. pseudomallei* infections in special populations and the possible association of melioidosis with novel immunosuppressive therapies and/or conditions require further investigation.

## 5. Co-Infections

Co-infection with melioidosis is uncommon but has been occasionally reported, particularly during outbreaks. These co-infections can complicate the clinical presentation and outcomes, making both diagnosis and treatment more challenging. Co-infections may involve viruses, bacteria, mycobacteria, or parasites. This section reviews some of the co-infections with *Burkholderia pseudomallei* and other microorganisms that have been reported in the literature to date.

### 5.1. Melioidosis and Viral Diseases

In endemic areas, dengue fever may coexist with melioidosis, complicating the diagnosis since both can present with fever, myalgia, and other nonspecific symptoms. A fatal case has been reported in Brazil in a young man with prolonged respiratory symptoms [82,83]. The inclusion of melioidosis in the reported differential diagnosis of community-acquired infections is mandatory in countries where both melioidosis and dengue fever are endemic because of the poor prognosis.

A case of melioidosis and Japanese encephalitis was reported in China in a 52-year-old man with fever, headache and respiratory symptoms. Neurological melioidosis is rare, accounting for only 5% of all melioidosis, and is therefore not often misdiagnosed. In this case, both JEV and *B. pseudomallei* infections were identified with different diagnostic tools in cerebrospinal fluid and blood [84].

The ongoing SARS-CoV-2 pandemic has also raised concerns about COVID-19 and melioidosis co-infections, particularly because both diseases can cause severe pneumonia and sepsis and could be especially severe in immunosuppressed patients. A nosocomial outbreak involving COVID-19 patients who developed acute melioidosis is further discussed below [7]. Another fatal case of reactivation of latent melioidosis associated with COVID-19 in a patient with risk factors has also been reported, as mentioned previously [67]. The reactivation of latent infections, such as melioidosis among immunosuppressed patients who develop COVID-19, should be considered.

Seasonal influenza may exacerbate the respiratory symptoms of melioidosis, leading to a more severe clinical course. A case of concurrent pulmonary melioidosis and influenza A in a pregnant woman has been described in Malaysia [85]. Influenza A is usually a self-limiting disease but is associated with high morbidity and mortality in high-risk populations, particularly during pregnancy. As both microorganisms can cause pneumonia, their diagnosis and management are challenging, and their prognosis may be poor without adequate antibiotic therapy. Influenza has also been associated with the reactivation of latent melioidosis, as illustrated by a report on a Vietnam veteran with sepsis following the activation of melioidosis in the setting of influenza [86].

### 5.2. Bacterial and Mycobacterial Co-Infections

Co-infection of tuberculosis and melioidosis is rare, with only 15 cases reported up to 2023, mostly in India [87] and the western Pacific region [88]. A mathematical estimate for Thailand of concurrent melioidosis and TB estimated an extremely low incidence of 0.0085 per 100,000 people [89]. Most of the notified cases have involved infections at the same anatomical site, primarily the lungs, though less common sites include cervical abscesses and lumbar spondylodiscitis. Only one case has been documented with simultaneous infections in different locations (pulmonary and digestive tuberculosis and a splenic abscess caused by *B. pseudomallei*) [88,90,91]. Diabetes mellitus is the most frequently identified risk factor, and there is a noted predominance of males among those affected [88]. In many cases, the co-infection was discovered incidentally through examination of respiratory samples, tissue biopsies, or blood samples [88].

Leptospirosis with melioidosis has been reported, mainly in Southeast Asia and northern Australia. Both diseases share similar environmental reservoirs, being associated with exposure to contaminated soil and water, especially during the rainy season. The biggest outbreak involving co-infection with these two bacteria has been described in a recreational forest in Malaysia (Lubuk Yu), where 153 people were exposed, including professional rescuers and villagers; 10 had a confirmed diagnosis of melioidosis, and 4 of those were co-infected with leptospirosis with a 70% of case fatality rate. Diabetes was a common risk factor among fatal cases [92,93]. Few other cases have been documented [94,95].

Complementary laboratory tests, including molecular tests [96], are essential for accurate diagnosis and management in cases where both infections may be involved and should include adequate coverage [95].

Cat scratch disease, caused by *Bartonella* spp., has become more frequent in certain geographical areas, especially among infants. A case of cat-scratch disease and melioidosis co-infection in a two-year-old boy with prolonged fever and painless cervical lymphadenitis has been described in Malaysia [97]. A history of travel to endemic areas and exposure to cats may be key clues to the diagnosis.

### 5.3. Parasitic Co-Infections

A report of a Sri Lankan female patient with diabetes mellitus, *B. pseudomallei* bacteremia and cutaneous leishmaniasis has been notified [98]. Although leishmania and melioidosis are common in this area, very few cases of co-infection have been described. A search for cases of melioidosis in association with other relevant parasitic diseases, such as malaria, strongyloidiasis, schistosomiasis and Chagas disease, did not identify additional cases.

The low frequency, as well as the clinical manifestations and risk factors common to some of these diseases, make the diagnosis of co-infections a great challenge, especially in non-endemic areas. However, given the overlapping distribution areas of many of these tropical diseases, the burden of co-infections in the context of melioidosis may well be unrecognized.

## 6. Laboratory Exposure and Nosocomial Transmission

Laboratory-acquired melioidosis is extremely rare. That said, *Burkholderia pseudomallei* in culture presents a risk to laboratory workers because of a low infectious dose coupled with the ease with which it is aerosolized [99]. Thus, it is paramount that if melioidosis is suspected clinically, the microbiology laboratory personnel must be notified to ensure that full laboratory safety measures are implemented [13]. There have been reports of laboratory personnel infected via a variety of clinical samples when this organism was unfortunately not suspected or misidentified initially [100,101]. Thus, it is recommended that work with *Burkholderia pseudomallei* be conducted in a biological safety cabinet, and gloves should always be worn when manipulating this organism [100]. In the event of a potential laboratory exposure, management guidelines have been suggested that include immediately washing any site of potential exposure with copious amounts of water, with the exposed worker immediately undergoing risk assessment to determine whether the laboratory incident poses a low risk or a high-risk exposure by appropriate occupational medical staff [100]. All those involved in high-risk incidents and low-risk incidents in those with risk factors for melioidosis should be offered PEP for 21 days with oral TMP-SMX, amoxicillin–clavulanic acid or doxycycline; however, the data for efficacy in humans are lacking [100]. Additionally, the exposed laboratory worker should self-monitor temperature for 21 days and be instructed to seek medical attention for any febrile illness with or without a cough [100]. Serologic testing after exposure, especially in non-endemic areas, should be considered [13,100]. Finally, all microbiologic laboratories, but especially those in non-endemic areas, should be aware of the growth characteristics of *B. pseudomallei* to minimize the potential for occupational exposure.

Exceptionally, nosocomial transmission of *B. pseudomallei* has also been described. The first two reported cases of hospital-acquired infection due to *Pseudomonas pseudomallei* were notified in 1979 when the organism was isolated from urine samples from catheterized diabetic patients in an endemic area of Australia [102]. Following this report, another case, which had been initially diagnosed in the early 1970s, involving a pulmonary infection acquired from a contaminated bronchoscope in Hawaii, was published [103]. A systematic review of neonatal melioidosis identified 22 cases, four of which were probable healthcare-associated infections (two of them fatal) and occurred in Thailand (n = 3) and Malaysia (n = 1) [43].

A cluster of six healthcare-associated cutaneous melioidosis cases due to contaminated wound irrigation fluid was also reported in a non-endemic area of Australia [104]. More recently, 25 patients with COVID-19 were diagnosed with acute melioidosis, mainly secondary bacterial pneumonia (in 88% of these patients), after admission to a COVID-19 field hospital in Thailand. In-hospital mortality for these co-infected patients was high (8/25, 32%). Genomic analysis of environmental samples testing positive for *B. pseudomallei* suggested contaminated tap water as the likely cause of illness [7].

Occupationally acquired melioidosis has also been described. Two mechanics in northern Australia with upper limb cutaneous melioidosis were probably infected following exposure to contaminated commercial hand wash detergent [105].

## 7. Imported Contaminated Products

Four cases of melioidosis, each identified in Georgia, Kansas, Minnesota and Texas, were found to be caused by a strain of *B. pseudomallei* associated with an imported aromatherapy spray. These isolates were clonal and matched an isolate from a bottle of room spray, Better Homes and Gardens Brand, that originated from India, a melioidosis-endemic area [106].

The patients included a 53-year-old woman with a history of chronic obstructive pulmonary disease, a 53-year-old man with alcohol dependence and tobacco use, and a 4-year-old girl and a 5-year-old boy without underlying health conditions. Their clinical courses ranged from pneumonia and bacteremia to septic shock; meningoencephalitis; nausea and vomiting; cerebral infarcts; osteomyelitis; osteonecrosis; and abscesses in lungs, liver and brain. Two died, and two had long-term debilitating sequelae. The aromatherapy spray from a patient was confirmed to have *Burkholderia pseudomallei* of the same strain [106].

Following the cluster of four melioidosis cases, the investigation discovered the aromatherapy spray to be a potential cause of animal disease [1]. The incident involved a previously healthy pet raccoon, owned by the family of the 5-year-old boy, who had broken a bottle of the aromatherapy spray and walked through the material. Two weeks after the exposure, the animal manifested acute neurologic symptoms and died 3 days later. The investigation tested multiple samples taken from the animal and the environment after these events, and two swabs collected from the raccoon’s intraorbital tissue tested positive by PCR for *B. pseudomallei,* although cultures did not yield viable *B. pseudomallei*. All other tissue samples tested negative by PCR or IHC, and no environmental contamination was detected by both PCR and culture. The outbreak-related strain found in the aromatherapy bottle contained a genetic variant, the bimA_Bm_ allele, which has been reported to be a virulence factor associated with neurologic melioidosis that is suspected to be the raccoon’s disease [1].

Another possible import-related exposure was described when a woman confirmed to have *B. pseudomallei* bacteremia was evaluated for possible environmental exposures. Three PCR-positive samples were obtained from her freshwater home aquarium with imported tropical fish, and whole-genome sequencing confirmed a genetic match with the patient’s clinical specimen. This incident raised the concern that the home aquarium materials or imported fish may be a source of melioidosis [107].

## 8. Imported Animals as Possible Sources

Melioidosis is not always considered a zoonosis; however, multiple animal species can be affected by *Burkholderia pseudomallei*. The trade of exotic animals has led to the emergence of cases in non-endemic regions [8,108]; this threat is ongoing, with cases recently being reported in non-human primates [109] and iguanas [110,111,112,113]. Animals believed to be less exotic may also contribute to melioidosis transmission; these include canines [114], ornamental fish [107,115] and goats [116]. Zoonotic transmission adds a layer of complexity to the Public Health response needed to control the emergence of melioidosis worldwide [108]. Recently, the first confirmed case of melioidosis in an animal in New Caledonia was identified. *B. pseudomallei* was isolated from a goat, suggesting possible human–animal transmission. The response involved analysis of the isolated strain using phylogenetics, which confirmed that the strain was related to those found in humans in the area. Recommendations were put in place to avoid further dissemination of the disease, including isolation of infected animals, use of PPE, clinical examination of the rest of the flock and culling of sick animals [116]. The potential zoonotic spread of melioidosis highlights the need for a One Health approach, including surveillance, increasing awareness of animal disease, improving diagnosis and access to diagnostic tools [116,117].

## 9. Conclusions and Future Perspective

Less than a decade ago, melioidosis was recognized as being endemic in 45 countries worldwide, but modeling studies suggested the disease may be endemic in at least another 30 countries, although a lack of systematic data in regions of South Asia, Africa and the Americas may be underestimating the true number of cases [2,3,108]. The burden of *B. pseudomallei* globally may, therefore, be greater than predicted. Rising temperatures, rainfall-related events and flooding have been linked with melioidosis, and environmental changes will likely contribute further to the geographical expansion of the disease [3,118].

An additional factor for consideration is the rise in type 2 diabetes observed in tropical countries, which may be undiagnosed or poorly controlled [119]. The majority of patients with diabetes live in low- and middle-income countries, and there is evidence that diabetes increases the severity of some endemic infections, such as melioidosis [119]. Thus, health services in tropical countries may suffer the double burden of infectious and non-communicable diseases [119].

**Figure 1:**
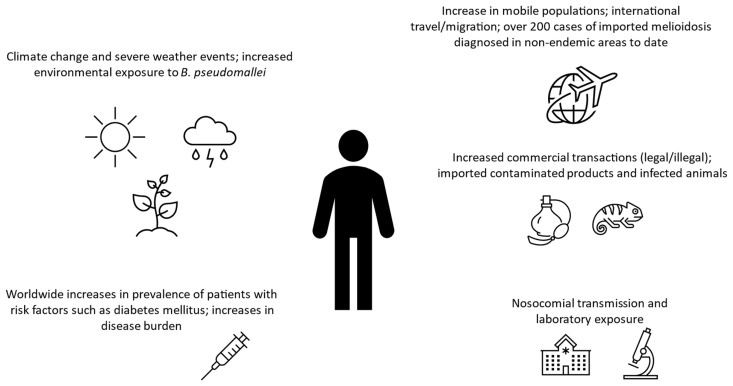
Factors that may contribute to recent changes in the global epidemiology of human melioidosis [4,5,7,8,9,30,99,106,108,119].

The future perspective for the evolving epidemiology of melioidosis arises in the context of globalization, influenced by population movements, climate change altering the ecological niches of *B. pseudomallei*, unusual epidemiological epidemic and pandemic situations, and technological advances in medicine paradoxically leading to a greater number of susceptible immunosuppressed or vulnerable patients. Figure 1 summarizes factors that may contribute to the recent changes in the global epidemiology of human melioidosis. All these intertwining factors can create challenges for the management of melioidosis but may also give rise to new opportunities for prevention and control.

## Figures and Tables

**Table 1:** Main characteristics of travel-associated melioidosis.

Previous Literature Reviews—Summary and Main Features										
Year Reference Number Cases (n)	Gender/Age	Country Exposure → Diagnosis	Main Risk Factors	Clinical Presentation	Diagnosis	Treatment	Time Return-Onset	Symptom Duration-Diagnosis	Duration Travel	Outcome Comments
Dan M, 2015 [9] n = 75 (1982–2015)	76.4% male Mean age 49.6 (4–90) years	Thailand (46%) Vietnam (8%) → Western Europe (69.5%)	Diabetes (21%) Chronic lung disease/ smoking (11%) Liver disease- alcoholism (7%)	Sepsis (34%) Pneumonia (29%) Abscess formation (25%)	NA	NA	Mean 3 weeks (range 0–52 weeks)	NA	Mean 36 days (range 7–330 days)	Mortality 17%
Norman et al., 2023 [8] n = 137 (2016–2022)	71% male Age range 15–83 years	Thailand (41%) India (9%)→ UK (40%) Netherlands (20%)	Diabetes (25%) Chronic lung disease (9%) Liver disease (5%)	Pneumonia (35%) Sepsis (30%) SSTI (14%)	NA	IV phase Ceftazidime (52%) Meropenem (41%) Continuation phase Co-trimoxazole (82%)	Less than 1 week (55%) More than 12 weeks (29%)	<1 week–18 months	Range from 9 days (travelers) to 10 years (expatriates)	Mortality 13%
Newly reported cases 2023–mid-2024										
Year Reference	Gender/Age	Country exposure → diagnosis	Risk factors	Clinical presentation	Diagnosis	Treatment	time return-onset	Symptom duration-diagnosis	Duration travel	Outcome Comments
Demas et al. 2023 [11]	Male 5th decade of life	Thailand→ France	Diabetes	Pneumonia Meningitis	BC: initially misidentified as *B. thailandensis* PCR confirmed *Burkholderia pseudomallei*	Amoxicillin Levofloxacin Meropenem −> Co-trimoxazole	NA	NA	NA	Survived
Igea et al. 2023 [10]	Male 48 years old	Mauritania→ Spain	Diabetes	Cavitated pneumonia Necrotic mediastinal lymph nodes Cutaneous abscesses Septic arthritis	BC, cutaneous abscess and joint fluid culture: *B. pseudomallei*	Meropenem + co-trimoxazole −> co-trimoxazole	4 weeks	1 week	NA	Survived
Hasani et al. 2024 [24]	Male 40 years old	Philippines→ UAE	Diabetes Alcohol use Animal farm	Sepsis Pneumonia Liver and prostatic abscess	BC: *B. pseudomallei*	Ceftazidime Meropenem + Co-trimoxazole−>co-trimoxazole	NA	NA	NA	Survived
Martinez et al. 2024 [25]	Male 67 years old	Honduras→ USA	Diabetes	Sepsis Altered mental status (encephalopathy) Liver abscess	BC *B. pseudomallei* (initially misidentified as *B. thailandensis*)	Meropenem −> Co-trimoxazole	Onset during travel	Less than 1 week	NA	Survived Laboratory exposures (prophylaxis offered, no seroconversions)
Gottschalk et al. 2024 [26]	Female 46 years old	Thailand→ Germany	No risk factors identified	Lung cavitation Mediastinal mass	Mediastinal lymphnodes PCR *B. pseudomallei* Positive serology	Ceftazidime Co-trimoxazole −> co-amoxiclav	3 weeks	4 weeks	6 months	Survived Rash with co-trimoxazole
Guldemond et al. 2024 [27]	Female 57 years old	Sri Lanka→ UK	Pre-diabetes Alpha thalassemia trait	Cerebral abscess	Cerebral abscess culture: *B. pseudomallei*	Meropenem + co-trimoxazole Ceftazidime + doxycycline	Onset during travel	10 weeks	NA	Survived Neutropenia with co-trimoxazole Deranged LFTs
Waters et al. 2024 [28]	Female 64 years old	India→ Canada	Diabetes Walked barefoot in brackish water (Chilika Lake)	Septic arthritis	Joint aspiration culture: initially misidentified as *B. thailandensis* PCR confirmed *B. pseudomallei*	Meropenem + co-trimoxazole Ceftazidime + co-trimoxazole−> co-trimoxazole doxycycline	6 weeks	Less than 1 week	NA	Survived Thrombocytopenia with co-trimoxazole
Balkhair et al. 2024 [30]	Male 65 years old	Thailand→ Oman	CKD Water splashes	Pneumonia Acute on chronic renal failure	BC: initially misidentified as *B. thailandensis* PCR confirmed as *B. pseudomallei* Sputum culture also positive	Ceftazidime + co-trimoxazole	Onset during travel	1 week	2 weeks	Survived
Im et al. 2024 [29]	Female 68 years old	Cambodia→ USA	Diabetes CKD Contact with animals (goat)	UTI	BC and urine culture: *B. pseudomallei*	Ceftazidime −> co-trimoxazole Doxycycline	NA	24 weeks	NA	Survived Pancytopenia with co-trimoxazole
Yuan et al. 2024 [23]	Male 56 years old	Myanmar −> China	Diabetes Metal mining	Pneumonia	BC WGS *B. pseudomallei* *BA*, *culture*, *WGS*: *B. pseudomallei*	Meropenem + levofloxacin	NA	16 weeks	NA	Survived

SSTI: skin and soft tissue infection; BC: blood cultures; PCR: Polymerase chain reaction; LFTs: liver function tests; CKD: chronic kidney disease; WGS: whole genome sequencing; BAL: bronchoalveolar lavage; NA: not available.
